# Peak transgene expression after intramuscular immunization of mice with adenovirus 26-based vector vaccines correlates with transgene-specific adaptive immune responses

**DOI:** 10.1371/journal.pone.0299215

**Published:** 2024-04-16

**Authors:** Sonia Marquez-Martinez, Nadine Salisch, Jan Serroyen, Roland Zahn, Selina Khan

**Affiliations:** Janssen Vaccines & Prevention B.V, Leiden, CN, The Netherlands; Fudan University, CHINA

## Abstract

Non-replicating adenovirus-based vectors have been broadly used for the development of prophylactic vaccines in humans and are licensed for COVID-19 and Ebola virus disease prevention. Adenovirus-based vectored vaccines encode for one or more disease specific transgenes with the aim to induce protective immunity against the target disease. The magnitude and duration of transgene expression of adenovirus 5- based vectors (human type C) in the host are key factors influencing antigen presentation and adaptive immune responses. Here we characterize the magnitude, duration, and organ biodistribution of transgene expression after single intramuscular administration of adenovirus 26-based vector vaccines in mice and evaluate the differences with adenovirus 5-based vector vaccine to understand if this is universally applicable across serotypes. We demonstrate a correlation between peak transgene expression early after adenovirus 26-based vaccination and transgene-specific cellular and humoral immune responses for a model antigen and SARS-CoV-2 spike protein, independent of innate immune activation. Notably, the memory immune response was similar in mice immunized with adenovirus 26-based vaccine and adenovirus 5-based vaccine, despite the latter inducing a higher peak of transgene expression early after immunization and a longer duration of transgene expression. Together these results provide further insights into the mode of action of adenovirus 26-based vector vaccines.

## Introduction

Non-replicating adenovirus-based vectors (AdV) have been extensively used for gene therapy and therapeutic vaccination, as well as prophylactic vaccines against infectious diseases that led to the licensed vaccines against COVID-19 disease (non-replicating adenovirus 26, adenovirus 5, and Y25-based vectors; Ad26, Ad5, ChAdOx1, respectively) and Ebola virus disease (Ad5 and Ad26 in combination with a Modified Vaccinia Ankara component) [1–5]. Adenoviruses are non-enveloped, double-stranded DNA viruses [6], and AdV vaccines have been engineered through genetic modifications that prevent viral replication, including deletions of the E1/3 regions of the adenoviral genome, creating space to insert a transgene of interest to induce an immune response against the transgene.

The development of transgene-specific adaptive immune responses is thought to be dependent on early events after vaccination such as AdV tropism, transgene expression and innate immune responses [7–9]. For instance, studies with Ad5 and other non-replicating adenovirus based vectors (Ad28, Ad35, chAd3, chAd63, sAd11, sAd16, ChadC68) have shown that the level and duration of transgene expression influences the maintenance and phenotype of cellular and/or humoral immune responses in mice [8–14]. However, there are few studies that address the direct relationship between early events and transgene-specific adaptive immune responses for other serotypes than adenovirus 5. One of these studies demonstrated that early termination of transgene expression in Ad5 immunized mice led to impaired memory CD8+ T cell responses [9]. At the same time, transgene expression is influenced by certain innate immune responses [8, 15], although the individual effect of transgene expression and innate immune responses on adaptive immune responses independently of each other has not been characterized.

While Ad26 vaccines have demonstrated to induce strong cellular and / or humoral immune responses against the transgene both in humans and preclinical animal species [16–19] a comprehensive understanding of the magnitude and duration of transgene expression after Ad26 vaccine dosing is limited [20]. These insights could lead to development of more immunogenic vectors through the modifications of the adenoviral particles, aiming to increase the magnitude of transgene expression by circumventing anti-viral innate sensing mechanisms [15] or retargeting transgene expression to more specific populations of antigen presenting cells [21, 22].

Here we characterized the magnitude and duration of transgene expression after a single intramuscular administration of Ad26 in mice and evaluated the differences to Ad5. We demonstrated higher peak transgene expression and duration of expression in mice immunized with Ad5 compared to Ad26. We showed that the magnitude of transgene expression early after Ad26 immunization correlates with transgene-specific cellular and humoral responses, while the difference in duration of transgene expression between Ad26 and Ad5 did not translate into differences in the magnitude of transgene-specific cellular memory responses.

## Results

### Magnitude and kinetics of transgene expression after intramuscular administration with AdV vaccines in mice

**Fig 1:**
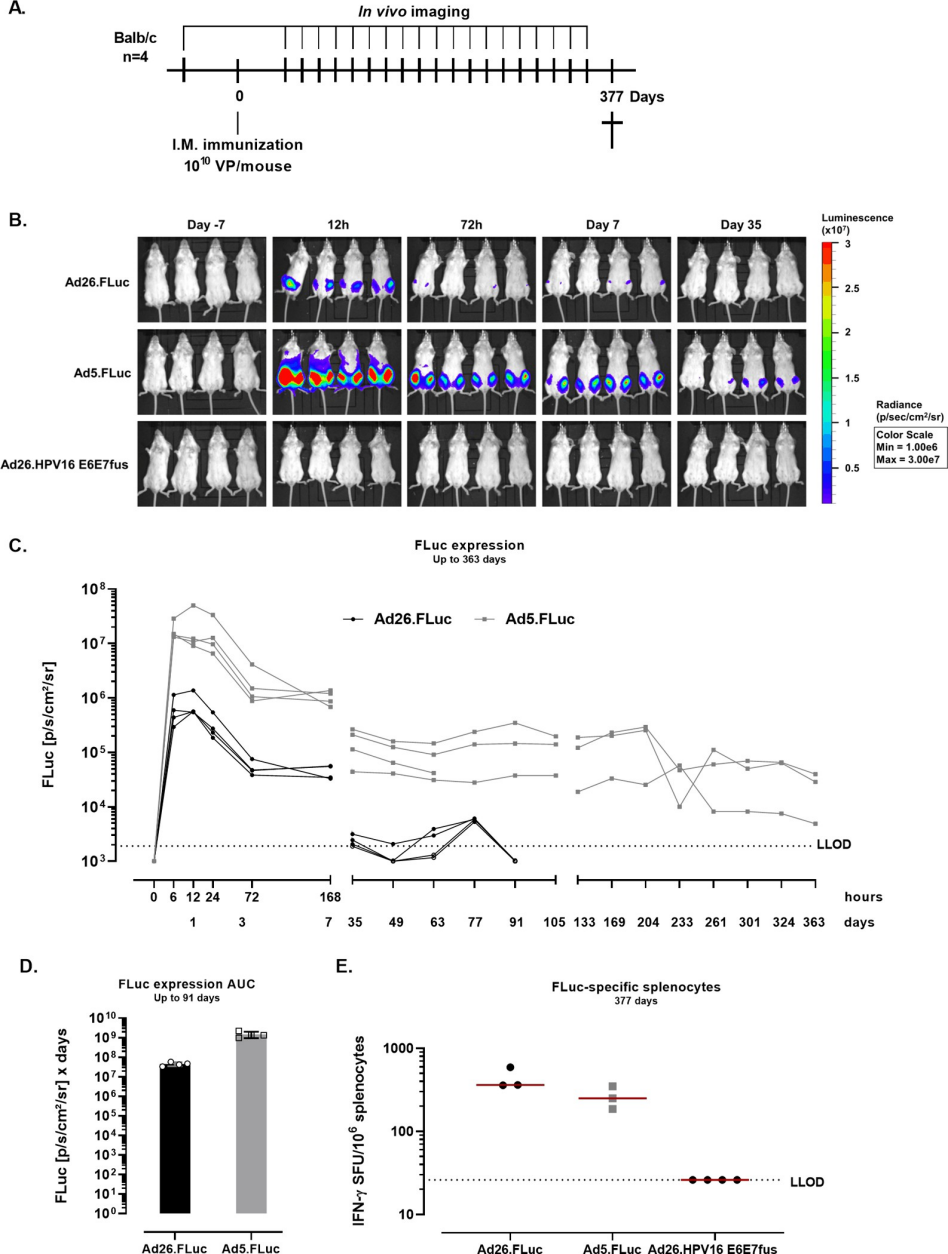
*In vivo* whole-body FLuc expression after AdV immunization in mice and FLuc-specific cellular responses. **A.** Experimental design. Balb/c mice (n = 4 per group) were dosed I.M. with 10^10^ VP/mouse of Ad26.FLuc, Ad5.FLuc, or Ad26.HPV16 E6E7fus (19), and FLuc signal was measured through *in vivo* bioluminescence imaging at different timepoints. **B.** Representative images of FLuc signal at different timepoints. **C.** Quantification of FLuc expression (photons per second per square centimeter per steradian, p/s/cm^2^/sr) after background subtraction (background = mean of signals measured in the Ad26.HPV16 E6E7fus group at the specific timepoints). The dashed line defines the lower limit of detection (LLOD) and corresponds to the average of the expression measured from the Ad26.HPV16 E6E7fus control group across timepoints + 3*STD **D.** Area under the curve (AUC) of the background subtracted measurements, up to day 91 **E.** FLuc-specific IFN-γ producing splenocytes (Spot forming units, SFU) were measured at day 377 after dosing. Splenocytes were stimulated with a peptide pool spanning the FLuc protein as described in the material and methods section. The dotted line indicates the background level (95th percentile of the medium stimulation). Datapoint from one mouse in the Ad26.FLuc group was not included due to a technical error in the ELISpot assay. One animal in the group dosed with Ad5.FLuc died during the course of the study (at day 77, FLuc expression data of this mouse is included up to day 63). Data were analyzed using a one-way ANOVA.

To understand the differences in the magnitude and kinetics of transgene expression, mice were immunized I.M. with 10^10^ adenoviral particles (VP)/mouse of Ad26 or Ad5 encoding firefly luciferase (FLuc) under a cytomegalovirus (CMV) promotor (Ad26.FLuc and Ad5.FLuc), or Ad26 encoding a human papillomavirus transgene (HPV16 E6E7fus) under a CMV promotor (negative control) and *in vivo* bioluminescent imaging (BLI) was conducted (Fig 1A). Residual FLuc protein was not detected in the vector batches confirming that all the measured FLuc signal came only from the transgene expression of the vector (S1 Fig). The FLuc signal was detected at 6h after dosing in Ad26.FLuc and Ad5.FLuc immunized mice and peaked within the first 24h after dosing. The highest signal was observed at the site of immunization (quadriceps) (Fig 1B), in all Ad5.FLuc and Ad26.FLuc dosed animals. The peak of FLuc signal was determined per animal (6h or 12h after dosing) and the magnitude of FLuc signal at the peak of expression was determined for the Ad26.FLuc (7.73x10^5^ p/s/cm^2^/sr ± 3.98x10^5^) and Ad5.FLuc (2.31x10^7^ p/s/cm^2^/sr ± 1.80x10^7^) groups, showing significantly higher magnitude in the Ad5.FLuc group (p = 0.0003, two-sample t-test). Notably, the FLuc signal was maintained for a year in Ad5.FLuc immunized mice, whereas the FLuc signal in Ad26.FLuc immunized mice was detectable until day 77 (Figs 1C and S2). The FLuc signal in the Ad26.FLuc group is considered positive until day 77 because there is detectable signal above LLOD in at least one mouse in all timepoints until day 77 and all mice in the group present signal above LLOD at day 77. An area under the curve analysis showed a 32-fold difference in the FLuc expression between Ad5.FLuc and Ad26.FLuc dosed animals (Fig 1D). Longer duration of FLuc expression in the Ad5.FLuc group did not lead to a statistically significant difference in the number of FLuc-specific IFN-γ producing cells one year after dosing compared with Ad26.FLuc induced cellular responses (p = 0.1019, ANOVA) (Fig 1E).

**Fig 2:**
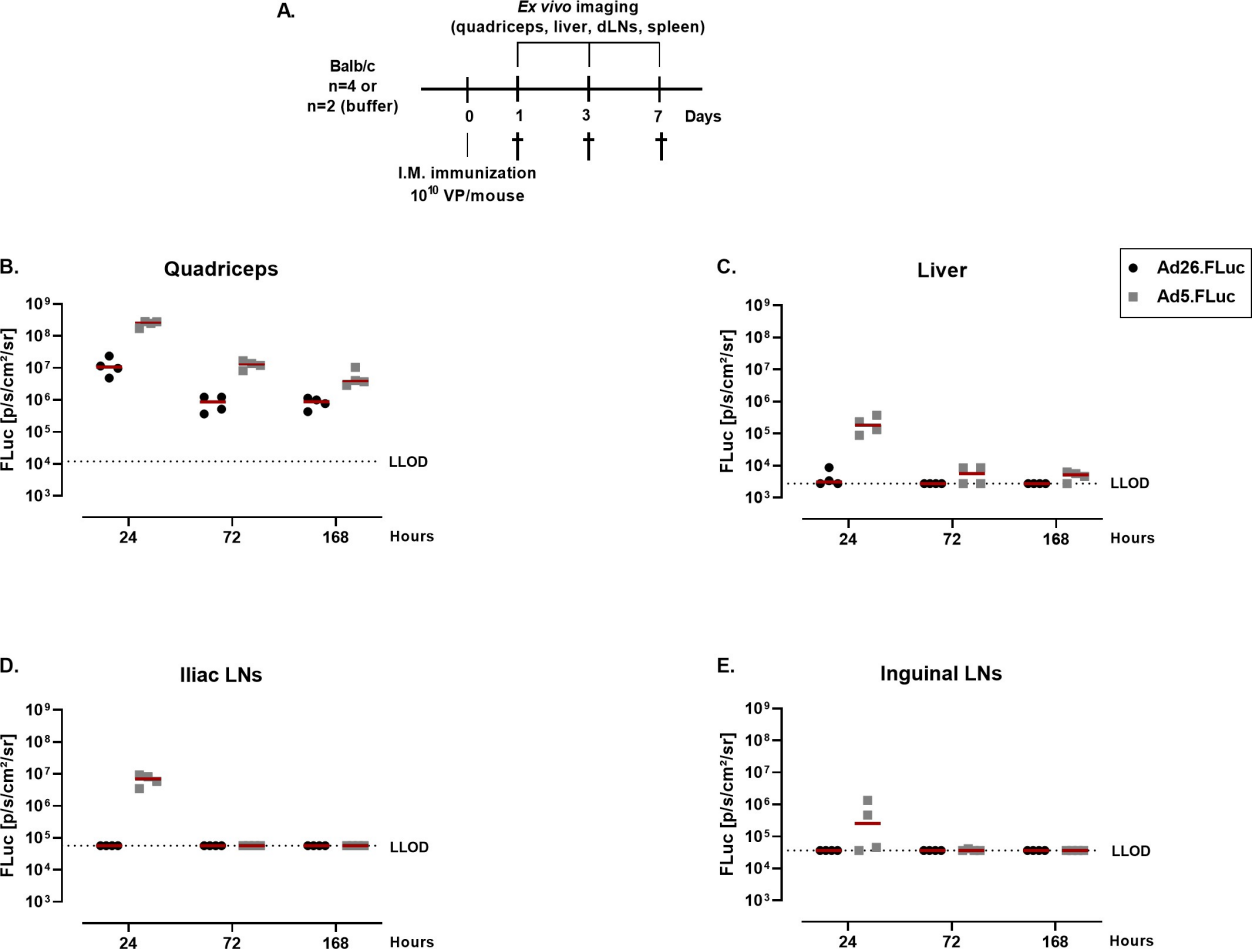
*Ex vivo* imaging of luciferase expression in organs of immunized mice. **A.** Experimental design. Balb/c mice (n = 4 study groups; n = 2 control group) were dosed I.M. with 10^10^ VP/mouse of Ad26.FLuc, Ad5.FLuc, or with saline buffer. Mice were sacrificed 24, 72 or 168hrs post dosing and **B.** Quadriceps **C.** Liver **D.** Iliac LNs **E.** Inguinal LNs were imaged *ex vivo*. Quantification of FLuc expression (p/s/cm^2^/sr) after background subtraction (background = mean of signals measured in the buffer group at the specific timepoints). The LLOD is defined is defined for each specific organ and corresponds to the average of the values from the saline group across timepoints + 3*STD of all values from the negative control.

To understand whether FLuc expression is limited to the site of immunization (hind legs, quadriceps), or it is distributed to other areas, mice were immunized with Ad26.FLuc or Ad5.FLuc at a dose of 10^10^ VP/mouse and quadriceps, draining lymph nodes (iliac and inguinal) and liver were removed directly after administration of luciferin to the mice at multiple timepoints after dosing (Fig 2A). The highest *ex vivo* FLuc signal was observed at the site of immunization (quadriceps) at all timepoints for Ad26.FLuc and Ad5.FLuc (Figs 2B and S3). In addition, the FLuc signal was detected in the draining lymph nodes (dLNs—inguinal and iliac) for Ad5.FLuc at 24h and rapidly waned to undetectable levels at 72h, while no signal was detected in the dLNs of Ad26.FLuc dosed animals at any timepoint (Fig 2D and 2E). The FLuc signal from Ad5.FLuc dosed animals was detectable in the liver with the highest expression observed at 24h in 4/4 mice (208155-fold above background) while a transient low signal was detected for Ad26.FLuc dosed animals at 24h in 2/4 mice (1.2-fold above background) (Fig 2C). At 72h after dosing, the signal was no longer detectable in the mice immunized with Ad26.FLuc, whereas a low signal was detected at 72h and 168h after immunization with Ad5.FLuc.

These *ex vivo* data confirm the *in vivo* biodistribution data showing that the FLuc expression for Ad26 and Ad5 peaks within the first 24h and wanes overtime and that Ad5 immunized mice present a higher FLuc signal.

### Correlation between peak transgene expression and transgene-specific immune responses after Ad26 intramuscular immunization

To understand whether transgene expression is a factor influencing adaptive immune responses after Ad26 vaccination, as has been described for Ad5 and AdC68 [9, 14], the correlation between the transgene-specific adaptive immune responses and the peak transgene expression in mice was assessed for two different antigens, FLuc (intracellular antigen) and SARS-CoV-2 Spike (membrane bound antigen).

**Fig 3:**
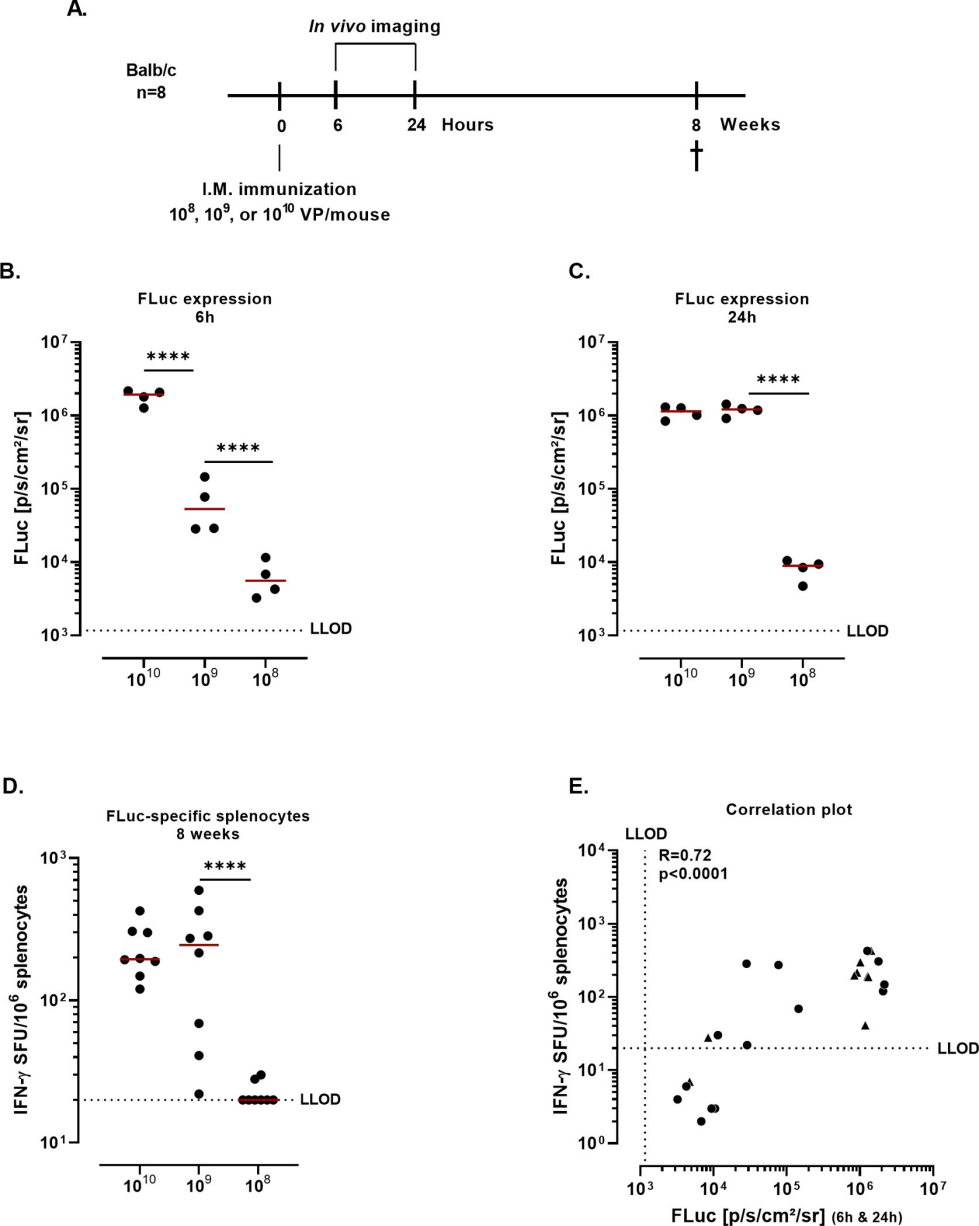
FLuc expression and FLuc-specific cellular responses in Ad26 immunized mice. **A.** Experimental design. Balb/c mice (n = 8/group) were dosed I.M. with 10^8^ VP/mouse, 10^9^ VP/mouse, or 10^10^ VP/mouse of Ad26.FLuc. Mice were injected subcutaneously with D-Luciferin at 6hr and 24h, and FLuc signal was measured through *in vivo* imaging **B.** Quantification of FLuc expression (p/s/cm^2^/sr) in half of the mice (n = 4) at 6h **C.** Quantification of FLuc expression (p/s/cm^2^/sr) (n = 4, not the same mice that were measured at 6h) at 24h. Data were analyzed using the Tobit model (**** = p<0.0001) and a correction for multiple comparisons was applied (Bonferroni). **D.** FLuc-specific IFN-γ producing splenocytes (SFU) were measured at 8 weeks after dosing (n = 8). Splenocytes were stimulated with a peptide pool spanning the FLuc protein. The dotted line indicates the background level (95^th^ percentile of the medium stimulation). Data were analyzed using the Tobit model (**** = p<0.0001) and a correction for multiple comparisons was applied (Bonferroni). E. Correlation analysis of FLuc expression and FLuc-specific IFN-γ producing splenocytes. Circles correspond to group for which FLuc expression was measured at 6h, triangles correspond to group for which FLuc expression was measured at 24h. Spearman correlation coefficient (R) and p-value (p) were calculated for the analysis.

Mice were immunized I.M. with Ad26.FLuc at increasing doses (10^8^, 10^9^, or 10^10^ VP/mouse) and the FLuc signal was measured in the timeframe of peak expression (at 6h or 24h after dosing) in two different groups of mice (Fig 3A). At 6h after dosing, FLuc signal showed a dose response pattern across dose levels (p<0.0001, Tobit model) (Fig 3B). At 24h after dosing, there was no difference in FLuc expression between the groups immunized with 10^10^ VP/mouse and 10^9^ VP/mouse, while the group immunized with 10^8^ VP/mouse presented lower levels of FLuc expression compared with the higher dose groups (p<0.0001, Tobit model) (Fig 3C). In line with this, the number of of FLuc-specific IFN-γ producing splenocytes responses was significantly lower at a dose of 10^8^ VP/mouse compared with a dose of 10^9^ VP/mouse (p<0.0001, Tobit model), while the numbers were comparable at doses 10^9^ and 10^10^ VP/mouse (Fig 3D). There was a strong correlation between the FLuc expression (at 6h and 24h after dosing) and FLuc-specific IFN-γ producing splenocytes (R = 0.72, p<0.0001, Spearman correlation) (Fig 3E). These results suggest a link between peak transgene expression and cellular responses after Ad26 vaccination in mice.

**Fig 4:**
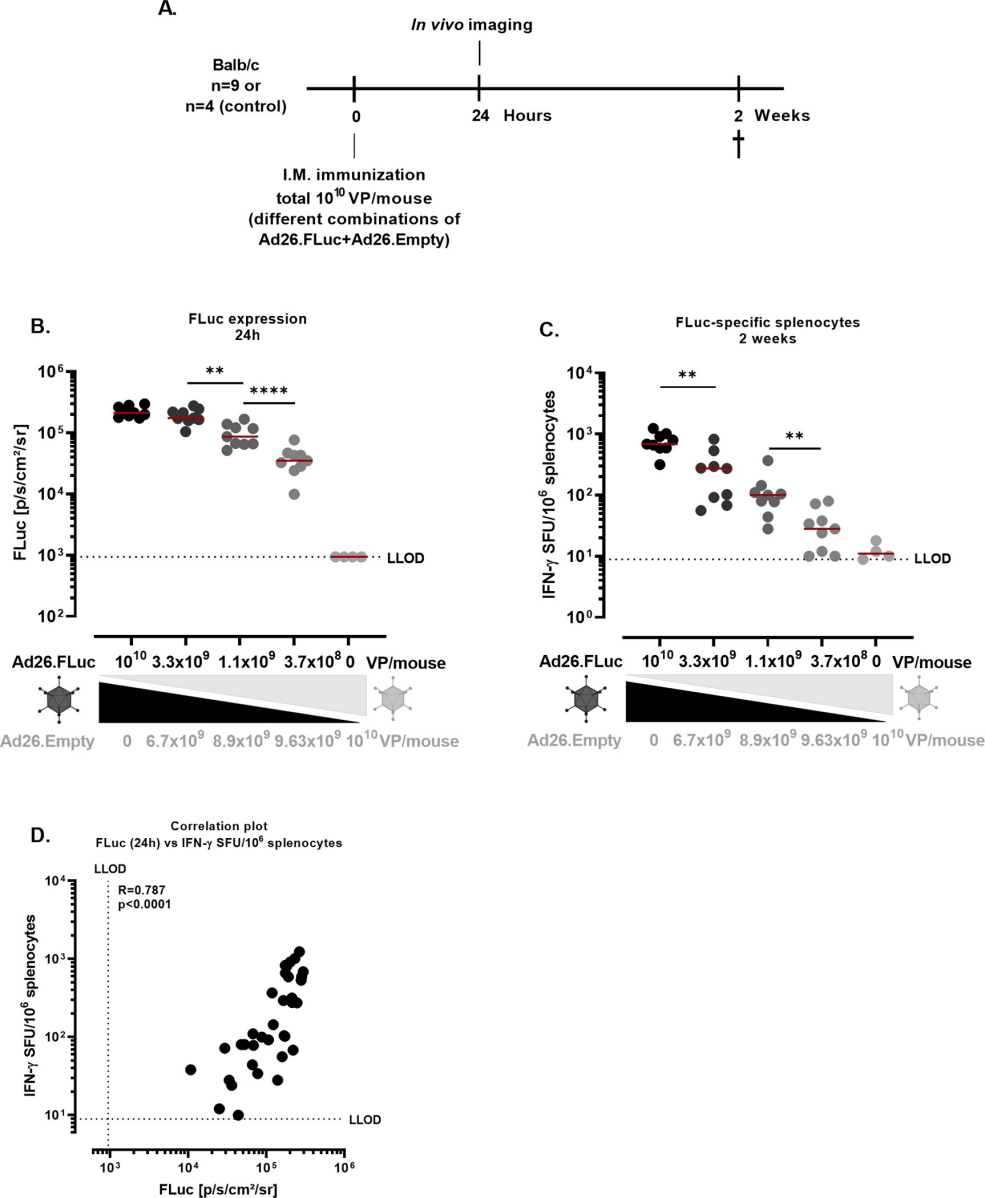
FLuc expression and FLuc-specific cellular responses in Ad26 immunized mice. **A.** Experimental design. Balb/c mice (n = 9/ study group; n = 4/ negative control group) were dosed I.M. with a total of 10^10^ VP/mouse, with decreasing concentrations of Ad26.FLuc and increasing concentrations of Ad26.Empty. Mice were injected subcutaneously with D-Luciferin 24h after dosing and FLuc signal was measured through *in vivo* imaging. **B.** Quantification of FLuc expression (p/s/cm^2^/sr) 24h after dosing after background subtraction (background = mean of signals measured in the Ad26.Empty group). The dashed line defines the lower limit of quantification (LLOD) and corresponds to the average of the expression measured from the Ad26.Empty control group across timepoints + 3*STD. Data were analyzed using one way ANOVA model (** = p<0.01; **** = p<0.0001) and a correction for multiple comparisons was applied (Bonferroni) **C.** FLuc-specific IFN-γ producing splenocytes (Spot forming units, SFU) were measured at 2 weeks after dosing. Splenocytes were stimulated with a peptide pool covering FLuc. The dotted line indicates the background level (95th percentile of the medium stimulation). Data were analyzed using a one-way ANOVA model (** = p<0.01) and a correction for multiple comparisons was applied (Bonferroni) **D.** Correlation analysis of FLuc signal and FLuc-specific IFN-γ producing splenocytes. Spearman correlation coefficient (R) and p-value (p) were calculated for the analysis.

However, groups that presented higher FLuc expression also received a higher vaccine dose (VP/mouse) than the other groups, precluding a conclusion on whether the observed difference is due to the higher number of VP/mouse leading to increased innate immune responses and thereby enhancing the priming, or due to the higher level of transgene expression as a result of the higher number of VP/mouse used. To address this, mice were immunized I.M. with a total dose of 10^10^ VP/mouse with various ratios of Ad26.FLuc and Ad26.Empty (Fig 4A). The FLuc signal was measured 24h after dosing (Fig 4B) and FLuc-specific IFN-γ producing splenocytes were measured by IFN-γ ELISpot 2 weeks after dosing (Fig 4C). Lower doses of the Ad26.FLuc vector resulted in decreased expression of FLuc as well as FLuc-specific cellular responses.

In alignment with the data shown in Fig 3, a strong correlation (R = 0.787, p<0.0001, Spearman correlation) was observed between transgene expression and transgene-specific IFN-γ producing splenocytes across all groups (Fig 4D), suggesting that transgene expression has a direct effect on transgene-specific cellular responses.

**Fig 5:**
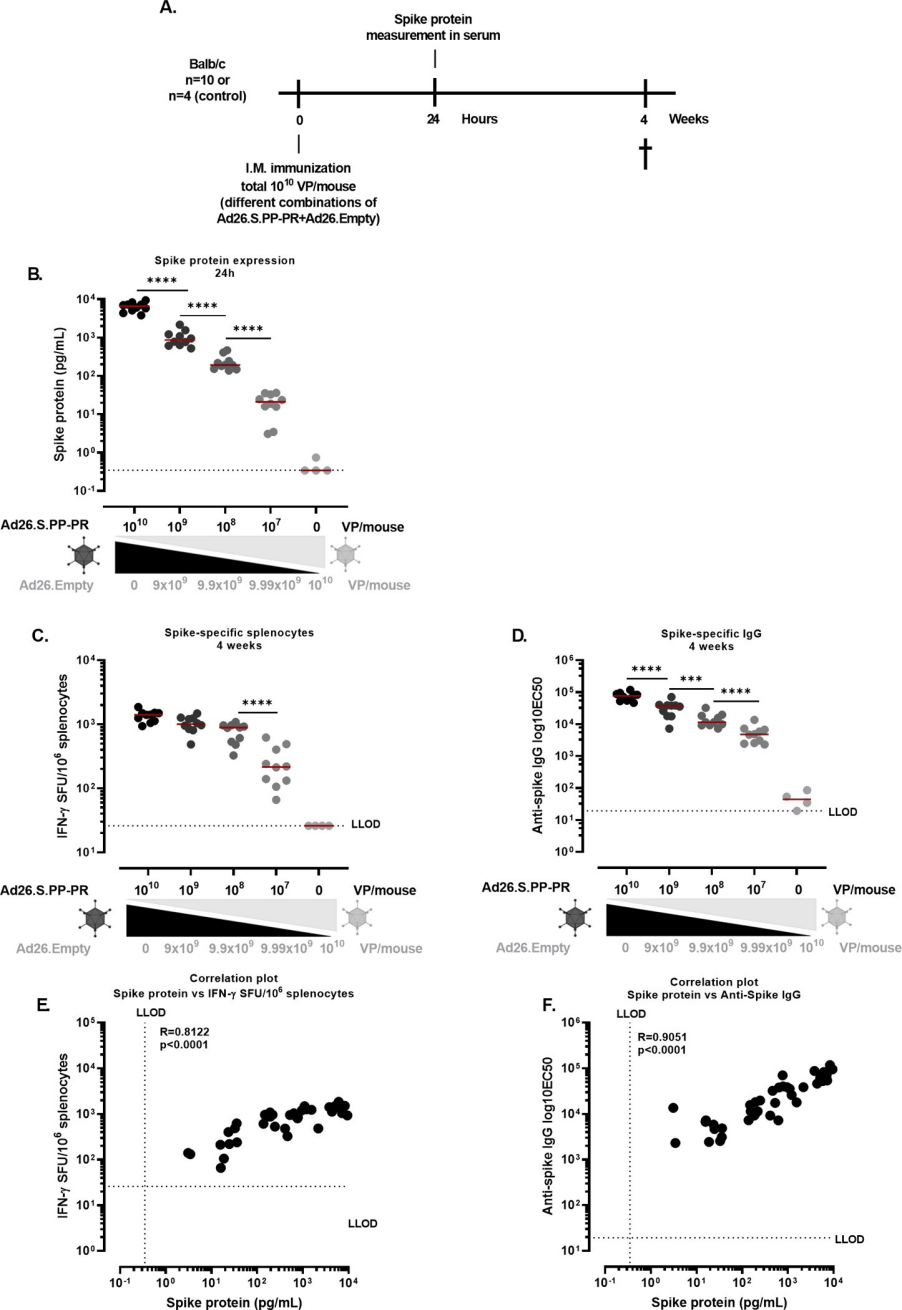
Spike protein expression and spike-specific cellular and humoral responses in Ad26 immunized mice. **A.** Experimental design. Balb/c mice (n = 10/ study group; n = 4/ negative control group) were dosed I.M. with a total of 10^10^ VP/mouse, with decreasing concentrations of Ad26.S.PP-PR and increasing concentrations of Ad26.Empty. **B.** Serum was collected at 24h after dosing and spike protein (picograms/milliliter, pg/mL) was measured in the serum through electrochemoluminescence. Data were analyzed using the Tobit model (**** = p<0.0001) and a correction for multiple comparisons was applied (Bonferroni). **C.** Spike-specific IFN-γ producing splenocytes (SFU) were measured at 4 weeks after dosing using IFN-γ ELISpot. Splenocytes were stimulated with a pool of peptides **of** the Spike protein (Wuhan strain). The dotted line indicates the background level (95th percentile of the medium stimulation). Data were analyzed using the Tobit model (**** = p<0.0001) and a correction for multiple comparisons was applied (Bonferroni). **D.** Spike-specific IgG (half maximal effective concentration, EC50) was measured in the serum at 4 weeks after dosing by ELISA. The dotted line indicates the LLOD of the assay. Data were analyzed using the Tobit model (*** = p<0.001; **** = p<0.0001) and a correction for multiple comparisons was applied (Bonferroni). **E.** Correlation analysis of spike protein expression and spike-specific IFN-γ producing splenocytes, and **F.** Correlation analysis of spike protein expression and spike-specific IgG titers. Spearman correlation coefficient (R) and p-value (p) were calculated for the analysis as described in the method section.

Certain AdVs have been reported to induce low levels of FLuc-specific antibody responses [13]. This could potentially be due to the intracellular nature and processing of the FLuc protein. Therefore, to assess whether transgene expression also correlates with transgene-specific humoral responses, a similar experiment using a SARS-CoV-2 spike transgene expressing Ad26 instead of Ad26.FLuc was performed. The Ad26.S.PP-PR vector used encodes a stabilized transmembrane spike protein with proline substitutions and a wild type furin cleavage site, as previously described [18]. Mice were immunized I.M. with a total dose of 10^10^ VP/mouse with various ratios of Ad26.S.PP-PR and Ad26.Empty (Fig 5A). Spike protein was measured in the serum (24h after dosing) (Fig 5B). A dose-response trend in spike expression was observed across all groups immunized with Ad26.S.PP-PR (p<0.0001, Tobit model in all comparisons) (Fig 5B). Spike-specific IFN-γ producing splenocytes and spike-specific antibodies were measured 4 weeks after dosing (Fig 5C and 5D). The number of spike-specific IFN-γ producing splenocytes were significantly higher in mice immunized with 10^8^ VP/mouse compared with the response seen at the 10^7^ VP/mouse (p<0.0001, Tobit model). There were no significant differences observed among the other groups. A dose-response trend in spike-specific IgG titers was observed in mice across all doses (10^10^, 10^9^, 10^8^ and 10^7^ VP/mouse of Ad26.S.PP-PR). Correlations were observed between spike protein expression and spike-specific IFN-γ producing splenocytes (R = 0.8122, p<0.0001, Spearman correlation) (Fig 5E) and between the spike-specific IgG titers and the spike protein expression (R = 0.9051, p<0.0001, Spearman correlation) (Fig 5F).

**Fig 6:**
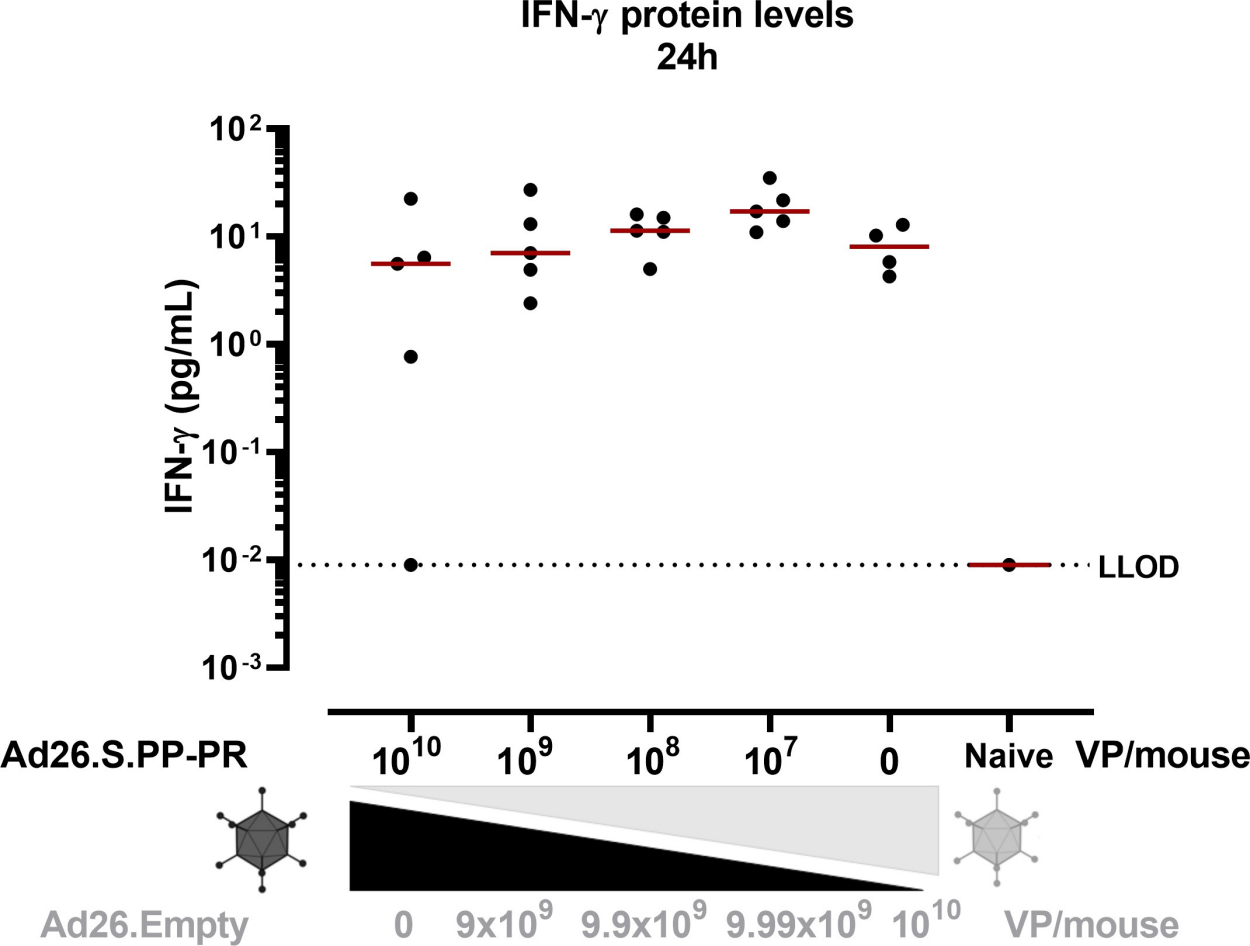
IFN-γ protein levels in serum of Ad26 immunized mice. Balb/c mice (n = 10/ study group; n = 4/ negative control group) were dosed I.M. with a total of 10^10^ VP/mouse, with decreasing concentrations of Ad26.S.PP-PR and increasing concentrations of Ad26.Empty. Serum was collected at 24h after dosing and IFN- γ levels were measured (picograms/milliliter, pg/mL) in randomly selected mice (n = 5/group) in a 1 in 10 dilution. In a separate run, Balb/c naive pool serum (dilution 1 in 10) was used to measure IFN-γ levels. Each symbol represents the average of 3 technical replicates. The dotted line indicates the LLOD of the assay defined as 2 standard deviations above background. Data were analyzed using the Tobit model and a correction for multiple comparisons was applied (Bonferroni).

IFN-γ expression in serum has been identified as a hallmark of innate immune activation 1 day after Ad26 immunization in non-human primates (NHPs) [15]. All study groups immunized with Ad26.S.PP-PR presented similar levels of IFN-γ in serum at 24h after dosing (Fig 6), indicating similar levels of innate immune activation across groups.

## Discussion

Non-replicating adenovirus-based vectors have been extensively used for gene therapy and therapeutic vaccination, as well as prophylactic vaccines against infectious diseases that led to the licensed vaccines against COVID-19 disease and Ebola virus disease [1–5]. The development of transgene-specific adaptive immune responses is dependent on early events after adenovirus-vector vaccination, such as transgene expression [8–10, 14], but there are few studies that address this question for other serotypes than Ad5. Here, we characterized the transgene expression and biodistribution after Ad26 vaccination and demonstrated a clear correlation between peak magnitude of transgene expression and transgene-specific immune responses in Ad26-immunized mice, independent of the dose of viral particles administered.

We observed transgene expression for up to 77 days after Ad26 immunization and >363 days after Ad5 immunization. Consistent with our data, dosing of Swiss Webster mice with Ad5 has resulted in duration of transgene expression (luciferase) for over 150 days [23]. These datasets conflict with a previously published study in which complete clearance of luciferase expression was observed by day 20 after Ad5 dosing [24]. This may be due to the use of the C57BL/6 mouse model compared with the Balb/c mouse model used in our studies, since it has been shown that the pigmentation of the C57BL/6 mouse skin attenuates bioluminescent signals [25]. Notably, faster clearance of the vector has been reported for other virus-based vaccine platforms, such as Modified Vaccinia virus Ankara (MVA) compared with AdVs in mice, with undetectable levels of the MVA vector at 72h after dosing [40]. RNA-based vaccines have been reported to express the SARS-COV-2 Spike transgene for over 9 days (mRNA) [41] or up to 63 days (saRNA) [26] in mice; and up to day 60 in humans [27]. This suggests that Ad26 is comparable to other vaccine platforms like saRNA in terms of vector clearance.

The lower magnitude and duration of transgene expression induced by Ad26 compared with Ad5 could be due to the cellular entry mechanisms or the innate immune responses triggered by the vector, which have been reported to play a role on the magnitude of transgene expression after AdV vaccination [21, 22, 28–32]. To this extent, Ad5 uses the coxsackie adenovirus receptor (CAR) to transduce cells [33], which is broadly expressed across tissues in mice (including endothelial and epithelial tissues) [34]; whereas Ad26 utilizes CD46 as the main receptor for transduction [35, 36], which is mainly restricted to the testis in mice [37], and sialic acids [38] and integrins [39] as alternative receptors. The broader receptor availability at the site of immunization and draining organs could lead to higher transduction rates in Ad5 immunized mice, explaining the higher magnitude of peak transgene expression. Moreover, the resolution of *in vivo* bioluminescent imaging does not allow to distinguish whether the transgene signal is at the site of immunization or at the draining lymph nodes, where Ad5 immune complexes could be retained for an extensive period of time in combination with follicular dendritic cells or other immune cells, as has been described for other antigens such as ovalbumin and B-Phycoerythrin [40, 41]. The retention of the antigen in the draining lymph nodes could explain the longer duration of transgene expression observed in Ad5 immunized mice.

Another potential factor that might explain the lower peak transgene expression induced by Ad26 compared with Ad5 is the anti-viral response triggered after cellular transduction. Ad5 virions undergo endosomal escape after cellular entry, whereas Ad26 virions accumulate in late endosomes, triggering innate responses that can lead to the destruction of the virions [42] and potentially prevent some of the adenoviral DNA from reaching the nucleus and producing transgene copies. Moreover, Ad26 vectors trigger the release of higher levels of pro-inflammatory cytokines (e.g. IFNα2, IFN-γ, IL-1β) in multiple species (mice, NHPs and human PBMCs) compared with Ad5 [15, 43], which may result in a faster clearance of the Ad26 vector. The precise innate responses that might influence transgene expression in mice after dosing with Ad26 remain to be further elucidated.

In our studies, Ad26 and Ad5 showed transgene expression at the site of immunization (quadriceps), whereas only Ad5 induced strong transgene expression in draining lymph nodes and liver, aligning with previous reports of transgene biodistribution in Ad5 immunized mice [24, 44]. It is important to note that the lack of detection of transgene expression in the draining lymph nodes of the Ad26 vaccinated animals in our studies might be due to limitations in the detection sensitivity. Transgene expression in the liver after Ad5 immunization (intravenous and intramuscular) of mice and rats has been previously reported [10, 24], but not after Ad26 immunization (intravenous or intramuscular) of mice [44]. Ad5 has been shown to transduce liver cells through factors IX [45] and X [44, 46] mediated CAR interactions, whereas these interactions have not been shown for Ad26 so far, explaining the low or undetectable signal in the liver of the mice immunized with Ad26 compared with Ad5. Interestingly, Ad5 has been reported to distribute to the liver and spleen but not to draining lymph nodes in rabbits [47] indicating either a lower sensitivity of the method used in this report (qPCR) or differences in the tropism of the transduced trafficked cells between species. The tropism of Ad5 may differ across species due to differences in the biodistribution of its primary cellular receptor CAR. For instance, CAR expression has been detected in human erythrocytes but not mouse erythrocytes [48]. Future studies should investigate the expression and biodistribution of the AdV primary receptors across species, and their involvement in transgene biodistribution and development of transgene-specific adaptive immune responses.

Despite the lower transgene expression, differences in transgene biodistribution and lower short-term transgene-specific immune responses observed in Ad26 immunized mice compared with Ad5, Ad26 induces robust T cell and antibody responses in preclinical models and humans. Although we did not perform a phenotypic characterization of the T cell responses in our studies, it has been described that Ad26 induces more polyfunctional transgene-specific T cell responses and enhanced memory T cell differentiation than Ad5 in mice [49]. Additionally, high levels of pre-existing anti-vector responses have been reported to impair immunogenicity against the transgene of interest in Ad5 vaccinees [50, 51]. Pre-existing Ad5 anti-vector immunity can lower vaccine effectiveness by blocking transduction and transgene expression [52]. However, a recent study assessed the influence of subsequent Ad26-based vaccination on transgene-specific immune responses in NHPs [17]. No clear consistent effect of pre-existing immunity was observed, aligning with the clinical data from homologous Ad26 or ChAdOx1 regimens showing consistent boosting after the second dose [16, 17, 53–58]. In addition, spike-expressing adenovirus-based vector vaccines Ad26.COV2.S and Ad5-nCoV have shown to elicit similar levels of neutralizing antibodies in humans [59].

Our studies showed that the dose-effect observed in transgene-specific adaptive immune responses after intramuscular one-dose immunization is tightly linked to the amount of transgene expressed, and not to the total number of adenoviral particles administered. These data suggest that co-stimulation of immune cells is directly dependent on the amount of transgene expression rather than on differences in pro-inflammatory cytokine levels. Specifically, transgene expression influences the potency of the cellular immune responses at least up to week 8 after dosing AdV vaccination, antigen duration beyond 77 days does not appear to improve the potency of the immune cellular response. This finding confirms the data reported by Finn et al., showing that termination of Ad5 transgene expression after 60 days does not influence CD8+ T cell memory maintenance [9]. Moreover, we show that the potency of transgene-specific T cell responses reaches a plateau at high doses of transgene-encoding adenoviral particles, suggesting there is a threshold in antigen expression after which cellular responses cannot be further enhanced, likely due to the saturation of antigen-loaded major histocompatibility complex class I (MHC-I). This plateau in transgene-specific T cell responses has been previously shown after spike expressing Ad26 vaccination in mice [18] and spike expressing mRNA vaccination in humans [60], indicating this may be the case for different platforms across species. In another study [61], peak transgene (FLuc) expression reached comparable levels across different platforms (Ad5, MVA, DNA and recombinant vaccinia virus (rVAC)) but Ad5 elicited the highest magnitude of cellular immune responses, suggesting that there are other factors aside from peak transgene expression that influence cellular responses.

B cell activation and antibody secretion is independent of antigen-loaded MHC-I molecules [62] and no plateau is observed in transgene-specific humoral responses in our studies or in previous reports after Ad26 or mRNA vaccination [18, 60]. Our findings suggest that the potency of humoral responses can be further enhanced through the increase of peak transgene expression. A correlation between spike-specific IgG titers and virus-neutralizing antibodies has been observed in previous studies in hamsters and NHPs [63, 64], therefore it is likely that peak transgene expression has a similar effect on virus-neutralizing antibodies. Importantly, humoral responses have been shown to correlate with protection against the disease caused by the Ebola virus in NHPs [65] and COVID-19 in human vaccinees [66, 67] after Ad26 administration, suggesting that an increase in the potency of humoral responses could lead to increased protection against disease. Previous reports have shown that repeated HIV protein-based vaccine administrations [68, 69] and sustained HIV antigen release through microneedle array implants [70] resulted in enhanced humoral responses compared to one dose administration due to the increased antigen availability during germinal center induction. The maintenance of transgene expression during germinal center induction is likely a key factor in the development of humoral responses after adenoviral-based vaccination. Modifications of the adenoviral particles that lead to higher peak transgene expression and maintenance could be key in the development of vaccines that elicit effective humoral responses and convey protection against the disease of interest.

Overall, our studies provide further insights in transgene expression and distribution, their effect on adaptive immune responses after Ad26 vaccination in a preclinical model, and the potential to increase the potency of transgene-specific humoral responses after AdV vaccination (and potentially other vaccine platforms) by increasing the magnitude of transgene expression.

## Material and methods

### Adenoviral vectors

E1/E3-deleted, replication-incompetent Ad26 or Ad5 vectors were engineered as described previously [71]. The FLuc (FLuc) transgene is based on an intracellular FLuc [71], the Human Papilloma Virus (HPV) transgene is a fusion protein of E6 and E7 of HPV16 [72] and the spike protein is a stabilized SARS-CoV-2 spike protein (S.PP-PR, [18]). The transgene identity was validated through PCR and sequencing of the products, and western blot analysis of infected A549 cell lysates or luciferase assay of infected A549 cells. The viral particle titers were measured by optical density at 260nm [73], and the infectivity was validated through TCID50 assay [74, 75]. The release criteria for animal experiments were met for bioburden and endotoxin levels.

### Animal experiments

Female Balb/c mice (specific pathogen-free), aged 5–12 weeks at the start of the study were purchased from Charles River laboratories (Sulzfeld, Germany). Mice were immunized with varying doses of Ad26.FLuc, Ad5.FLuc, Ad26.HPV16 E6E7fus, Ad26.S.PP-PR, or Ad26.Empty in 50 μl total volume of vaccine per hind leg under isoflurane anesthesia (I.M. immunization; see dosing in each individual figure).

Intermediate blood samples were collected via submandibular bleeding (at 24h after dosing, as indicated in figure). At the end of each study (see individual figures), animals were exsanguinated by heart puncture under anesthesia and sacrificed by cervical dislocation. Blood was processed for serum isolation and spleens were collected for humoral and cellular assays respectively. Mice experiments were designed according to the European guidelines (EU directive on animal testing 86/609/EEC) and Dutch legislation; and approved by the Central Authority for Scientific Procedures on Animals of the Netherlands (Centrale Commissie Dierproeven).

### *In vivo* imaging

Mice were immunized I.M. with different doses of Ad26.FLuc, Ad5.FLuc, Ad26.Empty or Ad26.HPV16 E6E7fus as indicated in the figure legends. At different timepoints, mice received 200μl of D-Luciferin Potassium Salt in PBS (15mg/mL) through subcutaneous administration in the scruff of the neck. After administration of luciferin, mice were kept awake for 5 minutes to allow distribution of the substrate before being imaged under anesthesia (isoflurane or ketamine/xylazine) using the IVIS Lumina II (Perkin Elmer). Regions of interest (ROI) were drawn for all animals covering the entire body for calculation of signal intensity. Light emission was measured in photons/s/cm^2^/sr (photon flux). Acquisition and analysis were performed with Living Image Software, Version 4.5 (Calliper LifeSciences, Hopkinton, MA).

### *Ex vivo* imaging

Mice were immunized I.M. with a dose of 10^10^ VP/mouse of Ad26.FLuc, Ad5.FLuc, or saline buffer. At 24h, 3 days and 7 days after dosing, mice received 200μl of D-Luciferin Potassium Salt as described above. Mice were kept awake for 5 minutes to allow biodistribution of the substrate and sacrificed through cervical dislocation. Organs were collected in buffer containing luciferin, ATP, and Mg^2^+ and imaged using the IVIS Lumina II (Perkin Elmer). Regions of interest (ROI) were drawn for all organs covering the entire organ for calculation of signal intensity. Light emission was measured in photons/s/cm^2^/sr (photon flux). Acquisition and analysis were performed with Living Image Software, Version 4.5 (Calliper LifeSciences, Hopkinton, MA).

### Luciferase detection in adenovirus vector batches

AdV batches (Ad26.FLuc, Ad5.FLuc, or Ad26.HPV16 E6E7fus) were diluted in buffer containing luciferin, ATP, and Mg^2^+ (30μl in 2mL of buffer). Luciferase protein (Sigma) was reconstituted in PBS (final concentration 1μg/μl), and 30μl were added to 2mL of buffer containing luciferin, ATP, and Mg^2^+ (positive control). The solutions were imaged using the IVIS Lumina II (Perkin Elmer). Light emission was measured in photons/s/cm^2^/sr (photon flux). Acquisition and analysis were performed with Living Image Software, Version 4.5 (Calliper LifeSciences, Hopkinton, MA).

### Peptide pools

For the studies in which FLuc antigen was used, a peptide pool composed of 15mer peptides overlapping by 4 amino acids spanning the FLuc sequence [76] was used in the IFN-γ ELISpot.

For the studies in which spike antigen was used, a peptide pool composed of 156 15-mers peptides overlapping by 11 amino acids **of** the SARS-CoV-2 Wuhan-Hu-1 (B) spike protein [77] was used in the IFN-γ ELISpot.

### IFN-γ ELISpot

Splenocytes were processed and IFN-γ producing cells specific for FLuc or spike were measured using a mouse IFN-γ ELISpot-plus kit (MabTech) as described previously [18]. Briefly, splenocytes were stimulated with the FLuc peptide pool (1 μg/peptide/mL, 0.4% DMSO), the spike peptide pool (1 μg/peptide/mL, 0.4% DMSO), or 0.4% DMSO in medium (negative control). All samples were run in duplicates. Plates were counted on an AELVIS ELISpot reader, and the numbers of spot-forming units (SFU) per 10^6^ cells were calculated. Background was defined as 95^th^ percentile of values from the 0.4% DMSO in medium.

### Detection of spike protein in serum by electrochemiluminescence

Complete EDTA-free protease inhibitor (Roche) was added to serum samples. Serum samples were centrifuged for 3 minutes, 2000x g. at 4°C to remove particulates before assay.

S-PLEX SARS-CoV-2 Spike detection assay (Mesoscale, detecting presence of the S protein RBD, direct communication from the manufacturer) was used to detect S protein in the serum samples, according to manufacture instruction, using PBS + 0.05% Tween-20 as washing buffer. All incubation steps were performed at 27°C.

The spike protein signal was measured using an MSD Sector S600 (model 1201) and the analysis was performed with the DISCOVERY WORKBENCH v4 software.

### Determination of spike-specific IgG in serum by ELISA

Total serum spike-binding IgG was measured by an ELISA. Briefly, ½ area 96-well OptiPlates (Perkin Elmer) were directly coated overnight at 4°C with SARS-CoV-2 S protein (COR200153, [63]) diluted in PBS at 2 μg/ml. Remaining S protein was removed and the plates were washed 3 times with PBS + 0.05% Tween-20 (PBS-T) and blocked with PBS 1% Casein for at least 1 hour at room temperature (RT), and then washed again.

Mouse serum was serially diluted (starting dilution 1:50) in sample buffer (PBS/1% Casein). Diluted samples were transferred to the coated Maxisorp 96-well ELISA plates (50μl/well in total), incubated for 60 minutes at RT and washed as described above. Bound IgG was detected with goat-anti-mouse IgG (H+L) conjugated to HRP (KLP/SeraCare) and detection substrate (electrochemiluminescence [ECL]) was added and incubated for 10 minutes. Luminescence was read on an BioTek Synergy Neo plate reader.

### Detection of IFN-γ in serum by ProQuantum ELISA

IFN-γ protein levels were measured in serum with a mouse IFN-γ ProQuantum ELISA detection assay (Thermofisher). The ProQuantum ELISA assay is based on antibody binding to the analyte that produces stabilized oligos that are amplified through qPCR. Serum was diluted 1 in 10 in assay dilution buffer and incubated with the antibody-conjugate mixture for 1h at RT. The qPCR protocol was performed according to manufacturer instruction. The qPCR was run in a ViiA 7 Real Time PCR Fast 96-well instrument. The data was analyzed with the ProQuantum software provided by the manufacturer.

### Statistical analysis

Data was log-transformed and groups were compared using a two-sample t-test or analysis of variance (ANOVA) in case of non-censored data, or a Tobit model in case of censored data. P-values <0.05 were considered statistically significant. A correction for multiple comparisons (Bonferroni adjustment) was applied where indicated. Correlation coefficients were calculated where indicated using the Spearman rank correlation.

## Supporting information

S1 Fig Residual FLuc protein in vector batches. Vector batches (Ad26.FLuc, Ad5.FLuc, or Ad26.HPV16 E6E7fus) (30 L/ batch) were diluted in 2mL of PBS and FLuc signal was measured through bioluminescence imaging. FLuc protein (30mg) was used as a positive control.
(TIF)

S2 Fig *In vivo* whole-body FLuc signal after AdV immunization in mice (late timepoints). FLuc signal from day 77 onwards from study shown in Fig 1. Balb/c mice (n = 4 per group) were dosed I.M. with 10^10^ VP/mouse of Ad26.FLuc, Ad5.FLuc, or Ad26.HPV16 E6E7fus (19), and FLuc signal was measured through *in vivo* bioluminescence imaging at different timepoints (77, 91 and 363 days after immunization). Empty square with diagonal line: data not available. One animal in the group dosed with Ad5.FLuc died during the course of the study (at day 77, FLuc expression data of this mouse is included up to and including day 63).
(TIF)

S3 Fig Representative images of luciferase expression in organs of immunized mice. FLuc signal from 24h from study shown in Fig 2. Mice were injected with D-Luciferin subcutaneously, sacrificed 24h after dosing and quadriceps, liver, iliac LNs and Inguinal LNs were collected. The organs were extracted and embedded in a buffer containing luciferin, ATP, and Mg^2+^; and FLuc signal was measured.
(TIF)
