# Supplementary figures and images for: Peak transgene expression after intramuscular immunization of mice with adenovirus 26-based vector vaccines correlates with transgene-specific adaptive immune responses
Source: PLoS One. 2024 Apr 16;19(4):e0299215. doi: 10.1371/journal.pone.0299215 (PMC11020485; doi:10.1371/journal.pone.0299215)

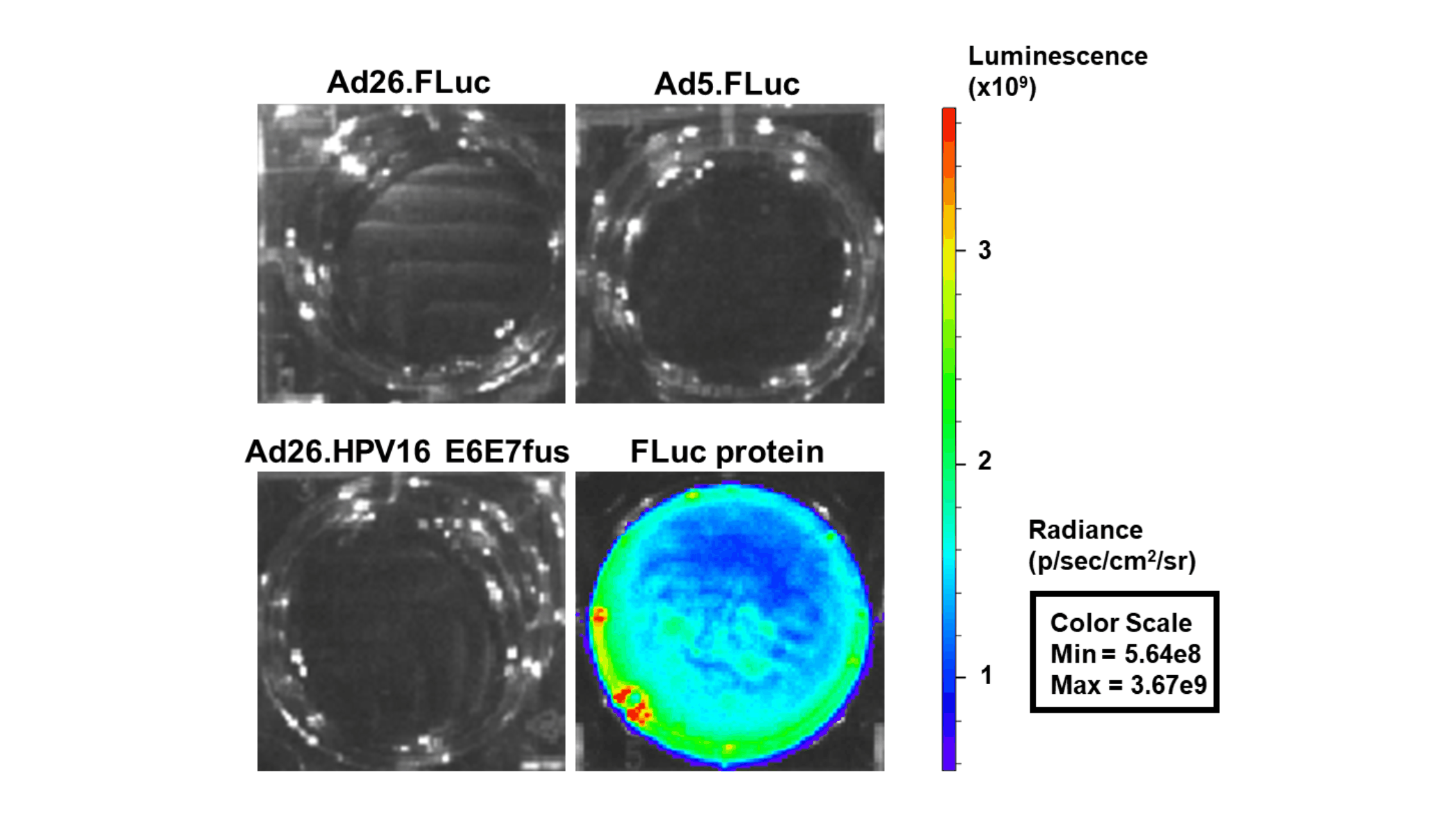

Supplement: S1 Fig — Vector batches (Ad26.FLuc, Ad5.FLuc, or Ad26.HPV16 E6E7fus) (30 L/ batch) were diluted in 2mL of PBS and FLuc signal was measured through bioluminescence imaging. FLuc protein (30mg) was used as a positive control. (TIF) [file pone.0299215.s001.tif]

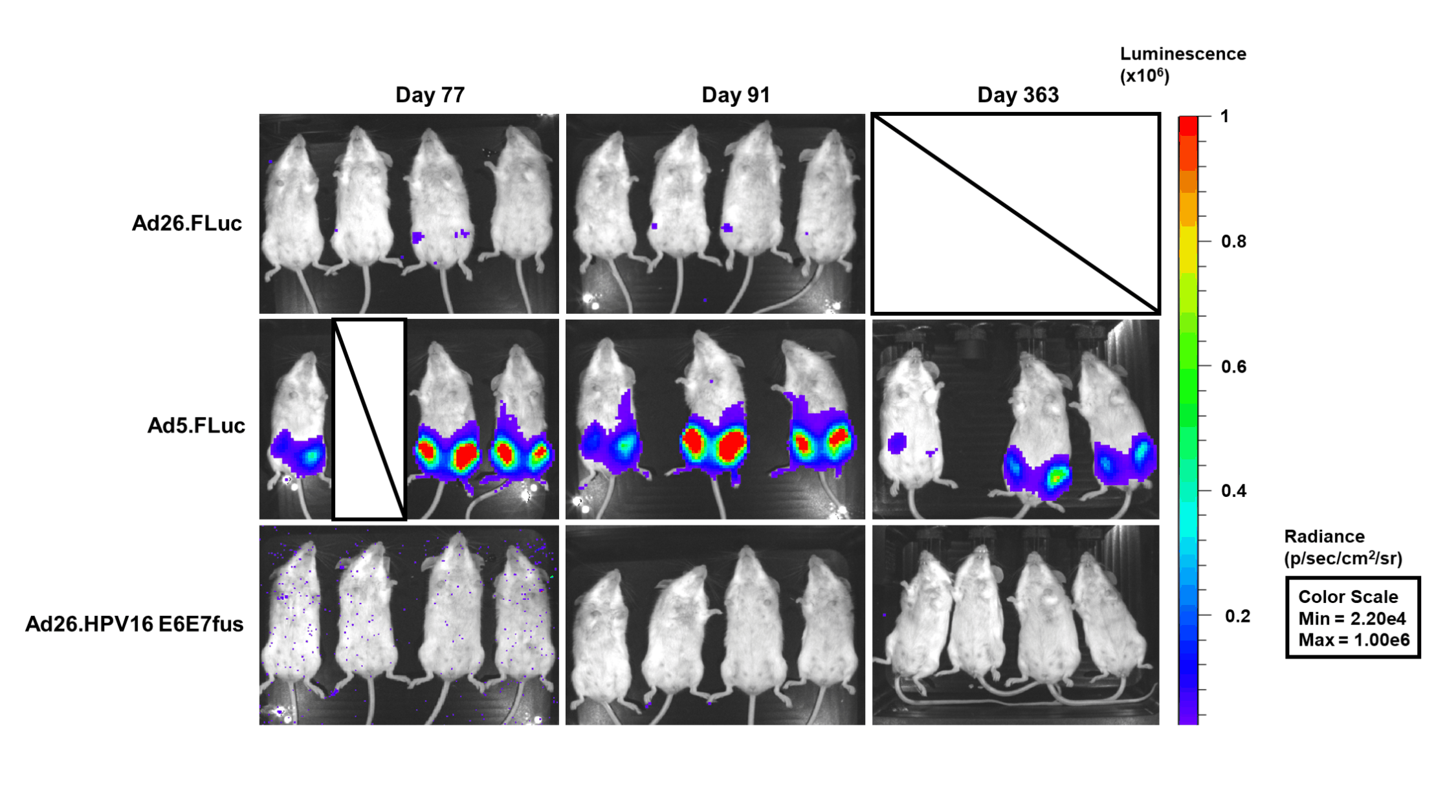

Supplement: S2 Fig — FLuc signal from day 77 onwards from study shown in Fig 1. Balb/c mice (n = 4 per group) were dosed I.M. with 1010 VP/mouse of Ad26.FLuc, Ad5.FLuc, or Ad26.HPV16 E6E7fus (19), and FLuc signal was measured through in vivo bioluminescence imaging at different timepoints (77, 91 and 363 days after immunization). Empty square with diagonal line: data not available. One animal in the group dosed with Ad5.FLuc died during the course of the study (at day 77, FLuc expression data of this mouse is included up to and including day 63). (TIF) [file pone.0299215.s002.tif]

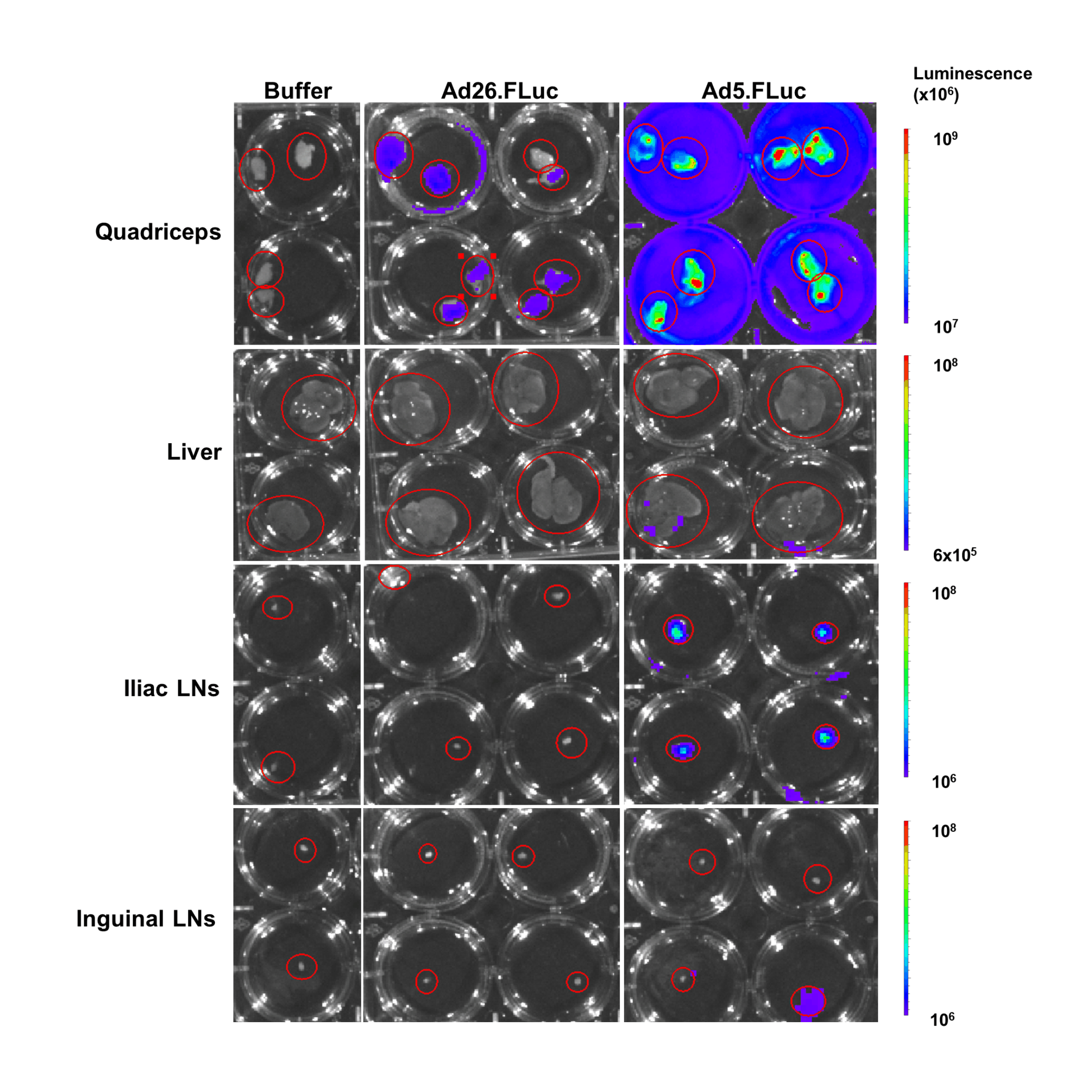

Supplement: S3 Fig — FLuc signal from 24h from study shown in Fig 2. Mice were injected with D-Luciferin subcutaneously, sacrificed 24h after dosing and quadriceps, liver, iliac LNs and Inguinal LNs were collected. The organs were extracted and embedded in a buffer containing luciferin, ATP, and Mg2+; and FLuc signal was measured. (TIF) [file pone.0299215.s003.tif]
